# Phase 1b/2 study of ibrutinib and lenalidomide with dose-adjusted EPOCH-R in patients with relapsed/refractory diffuse large B-cell lymphoma*

**DOI:** 10.1080/10428194.2021.1907371

**Published:** 2021-04-15

**Authors:** Wyndham H. Wilson, Tycel Phillips, Leslie Popplewell, Sven de Vos, Saurabh Chhabra, Amy S. Kimball, Darrin Beaupre, Da Wei Huang, George Wright, Kevin Kwei, Jerry Ping, Jutta K. Neuenburg, Louis M. Staudt

**Affiliations:** a Lymphoma Therapeutics Section, National Cancer Institute, Bethesda, MD, USA; b Division of Hematology and Oncology, University of Michigan, Ann Arbor, MI, USA; c Department of Hematology and Hematopoietic Cell Transplantation, City of Hope National Medical Center, Duarte, CA, USA; d Department of Medicine, David Geffen School of Medicine at UCLA, Los Angeles, CA, USA; e Department of Medicine, Medical College of Wisconsin, Milwaukee, WI, USA; f University of Maryland School of Medicine, Baltimore, MD, USA; g Early Development and Immunotherapy, Pharmacyclics LLC, an AbbVie Company, Sunnyvale, CA, USA; h Department of Translational Medicine, Pharmacyclics LLC, an AbbVie Company, Sunnyvale, CA, USA; i Department of Statistics, Pharmacyclics LLC, an AbbVie Company, Sunnyvale, CA, USA; j Department of Oncology, Pharmacyclics LLC, an AbbVie Company, Sunnyvale, CA, USA; k Lymphoid Malignancies Branch, National Cancer Institute, Bethesda, MD, USA

**Keywords:** Ibrutinib, lenalidomide, dose-adjusted EPOCH-R, diffuse large B-cell lymphoma, activated B-cell-like, germinal center B-cell-like

## Abstract

Relapsed/refractory diffuse large B-cell lymphoma (DLBCL) is difficult to cure; non-germinal center B-cell-like (non-GCB) and activated B-cell-like (ABC) DLBCL have worse outcomes than GCB DLBCL. Ibrutinib and lenalidomide are synergistic in vitro in ABC DLBCL and may augment salvage chemotherapy. In part 1 of this phase 1b/2 study (NCT02142049), patients with relapsed/refractory DLBCL received ibrutinib 560 mg and escalating doses of lenalidomide on Days 1–7 with DA-EPOCH-R (Days 1–5) in 21-day cycles. In part 1 (*N* = 15), the maximum tolerated dose was not reached with lenalidomide 25 mg (recommended part 2 dose [RP2D]); most common grade ≥3 adverse events were anemia (73%) and febrile neutropenia (47%); the overall response rate (ORR) was 40%. At the RP2D (*n* = 26), ORR was 71% in non-GCB and 64% in ABC. Ibrutinib and lenalidomide with DA-EPOCH-R had a manageable safety profile and antitumor activity in relapsed/refractory DLBCL, especially the non-GCB subtype.

## Introduction

Diffuse large B-cell lymphoma (DLBCL) is the most common subtype of non-Hodgkin lymphoma in the USA [1]. DLBCL includes two major subtypes: non-germinal center B-cell-like (non-GCB), including activated B-cell-like (ABC) DLBCL, and germinal center B-cell-like (GCB) DLBCL [2]. Recommended first-line therapy for DLBCL is rituximab, cyclophosphamide, doxorubicin, vincristine, and prednisone (R-CHOP) [3,4] or dose-adjusted etoposide,prednisone, vincristine, cyclophosphamide, doxorubicin, and rituximab (DA-EPOCH-R) for patients with high-risk features [5–7]. While ~60% of patients are cured by standard therapy [8], prognosis is poor among patients who are refractory or who relapse early [9,10]. Second-line salvage treatments for relapsed/refractory (R/R) DLBCL include rituximab plus ifosfamide, carboplatin, and etoposide (R-ICE) [10,11]; rituximab plus dexamethasone, cisplatin, and cytarabine [10,12]; and DA-EPOCH-R [13,14]. CAR T cells, polatuzumab, selinexor, and MOR208 have recently become available [15,16], and ibrutinib [17] and lenalidomide [18,19] for non-GCB DLBCL.

B-cell receptor (BCR) signaling is a pathogenic mechanism of ABC DLBCL, activating the NF-κB pathway, and potentially increasing chemotherapy resistance [20]. Bruton’s tyrosine kinase inhibition blocks downstream BCR signaling and prevents B-cell proliferation [21]. Ibrutinib, a once-daily Bruton’s tyrosine kinase inhibitor, is approved in the United States for various B-cell malignancies. Single-agent ibrutinib and ibrutinib-based combinations are active in R/R DLBCL, especially the ABC subtype [17]. Results of a phase 1/2 study of single-agent ibrutinib showed an overall response rate (ORR) of 37% (complete response [CR], 16%) in R/R ABC DLBCL (*n* = 38) and 5% in GCB DLBCL (*n* = 20) [17]. Median overall survival (OS) was 10.32 months in ABC and 3.35 months in GCB DLBCL [17]. Furthermore, ibrutinib plus R-ICE showed activity and tolerability in a phase 1 dose-escalation study of R/R DLBCL, with ORRs of 90% overall and 100% in the non-GCB subtype [22]. In the phase 3 PHOENIX study (NCT01855750; *N* = 838), ibrutinib plus R-CHOP versus R-CHOP alone showed increased toxicity but significantly improved event-free survival and OS in patients <60 years of age [23]. There was no benefit in patients ≥60 years, likely due to a higher rate of R-CHOP discontinuations and reduced ibrutinib exposure compared to patients <60 years.

Lenalidomide, an immunomodulatory drug, has demonstrated single-agent activity in R/R DLBCL, especially the non-GCB subtype [18]. ORRs of 52.9% (CR, 29.4%) and 8.7% (CR, 4.3%) were achieved in lenalidomide-treated patients with non-GCB (*n* = 17) and GCB (*n* = 23) subtypes, respectively; median progression-free survival (PFS) was 6.2 and 1.7 months [18]. However, lenalidomide combined with R-CHOP did not significantly prolong PFS versus R-CHOP alone (hazard ratio, 0.85; 95% confidence interval [CI], 0.63–1.14; *p* = 0.29) in first-line ABC DLBCL [24].

Ibrutinib and lenalidomide may augment the activity of DA-EPOCH-R via inhibition of BCR signaling and NF-κB activation, as both are drivers in non-GCB and ABC DLBCL; thus, the combination of DA-EPOCH-R, ibrutinib, and lenalidomide was evaluated in R/R DLBCL.

## Methods

### Patients and treatment

**Figure 1.**
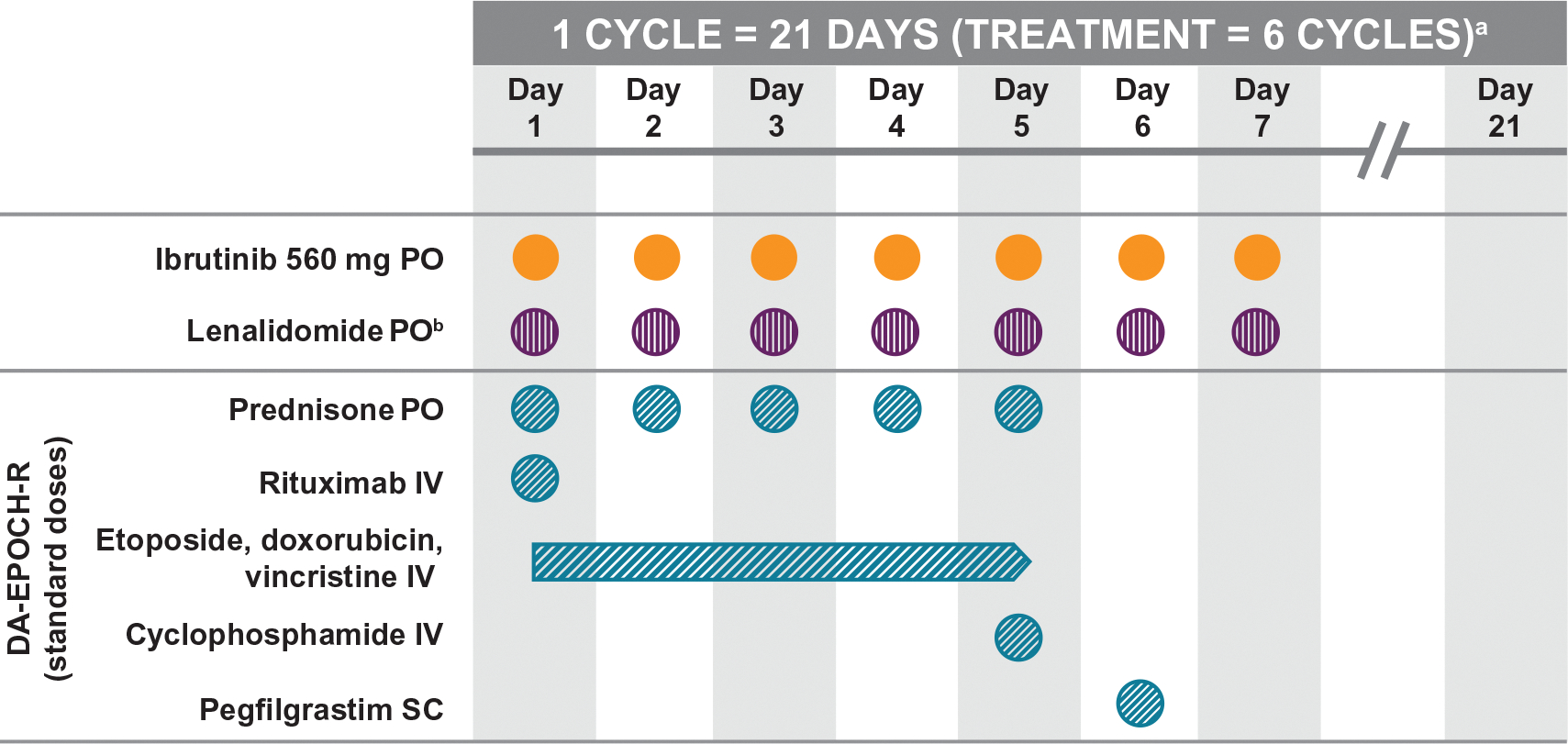
Study schema. DA-EPOCH-R: dose-adjusted cyclophosphamide, doxorubicin, etoposide, vincristine, and prednisone, with or without rituximab; IV: intravenous; PO: orally; SC: subcutaneous. ^a^Ibrutinib and lenalidomide were administered on days 1–7 of a 21-day cycle for up to six cycles. ^b^Dose escalated at doses of 0, 15, 20, and 25 mg (dose levels 1, 2, 3, and 4, respectively). Dose-limiting toxicity was assessed during the first treatment cycle.

This phase 1b/2, nonrandomized, multicenter, open-label study enrolled patients with R/R DLBCL (NCT02142049). The study was conducted in two parts. Part 1 used a standard 3 + 3 design to determine the maximum tolerated dose (MTD) of the ibrutinib, lenalidomide, and DA-EPOCH-R combination. Patients received a fixed dose of ibrutinib (560 mg) and lenalidomide (escalating doses: 0 [level 1], 15 [level 2], 20 [level 3], and 25 [level 4] mg) on Days 1–7 of each 21-day cycle and standard doses of DA-EPOCH-R on Days 1–5 of each 21-day cycle (Figure 1). Part 2 evaluated therapeutic activity and safety. The lenalidomide dose was to be adjusted for hematologic toxicities (Supplementary Text S1).

Adults ≥18 years of age with pathologically confirmed, measurable DLBCL were eligible for the study. Part 1 enrolled patients with all subtypes of DLBCL; part 2 enrolled patients with the non-GCB subtype as determined by immunohistochemistry (IHC) using the Hans criteria. Patients had R/R disease defined as recurrence of disease after CR or disease progression, an Eastern Cooperative Oncology Group performance status of ≤2, and left ventricular ejection fraction >45%. Patients with transformed DLBCL, coexisting histologies, or primary mediastinal lymphoma were eligible for part 1 only. Patients with an allogeneic stem cell transplant within 6 months of study start were ineligible. Patients did not receive prophylactic antibiotics. This study was conducted in accordance with the ethical principles of the Declaration of Helsinki, consistent with Good Clinical Practices and applicable regulatory requirements. All patients provided written informed consent.

### Endpoints and assessments

Part 1 primary endpoints were MTD and safety and tolerability in R/R DLBCL; the secondary endpoint was ORR (CR plus partial response [PR]). The part 2 primary endpoint was ORR in ABC DLBCL (subtype determined per gene expression profile [GEP]); secondary endpoints were duration of response (DOR), PFS, OS, and safety and tolerability in R/R ABC DLBCL. Exploratory endpoints were pharmacokinetics and biomarkers of ibrutinib sensitivity or resistance.

Response assessments were performed per investigator according to the revised International Working Group Response Criteria for non-Hodgkin lymphoma [25]. Computed tomography and positron-emission tomography scans were required for pretreatment assessment within 21 days of first dose and for CR (Supplementary Text S2). All adverse events (AEs) were recorded from first dose until 30 days after last dose of study drug.

Pharmacokinetic analyses were conducted in 15/20 patients in part 2 only. Blood samples were collected pre-dose and at 1, 2, 4, and 6 h after ibrutinib administration on Cycle 1 Day 5. Plasma samples were analyzed for ibrutinib and PCI-45227 (metabolite) concentrations by Frontage Laboratories (Exton, PA, USA); plasma concentration was analyzed by noncompartmental methods using validated Phoenix WinNonlin software (version 6.3). *C*_max_ and *T*_max_ were recorded; apparent *t*_1/2_ and AUC_0–24h_ were calculated using ln_2_/*λ*_z_.

For eligibility in part 2, patients were identified as GCB or non-GCB via IHC using Hans criteria [26]. For the primary analysis in part 2, ABC DLBCL subtype was determined by GEP. Sufficient biopsy material was available for molecular analysis for 31 patients. Biopsies were studied using the Lymph2CX 20-gene GEP assay (NanoString Technologies, Seattle, WA, USA) [27]. Of 31 samples, 30 had sufficient material for additional analyses, including RNA sequencing (*n* = 28 [2 sequencing failures]) and/or whole exome sequencing (WES; *n* = 15 [insufficient DNA/sample quality precluded WES, *n* = 15]); 13 cases were studied by RNA sequencing and WES. Cell-of-origin assignment (ABC, GCB, or unclassified) was based on consensus RNA sequencing and NanoString calls [28]. Mutations were called using the WES and/or RNA-sequencing aligned reads [28]. Data were filtered to remove any mutation reported in dbSNP (https://www.ncbi.nlm.nih.gov/snp) or EXAC database (prevalence ≥0.0001; http://exac.broadinstitute.org). Tumor samples were assessed by PhenoPath Laboratories (Seattle, WA, USA) to determine tumor content. If <50% tumor content was present on formalin-fixed paraffin-embedded slides, samples were macrodissected to enrich for tumor material (Supplementary Text S3).

### Statistical methods

The all-treated population included enrolled patients who received ≥1 dose of any study drug and was used to analyze activity and safety endpoints in part 1 and for patients treated at the recommended part 2 dose (RP2D) in parts 1 and 2. The response-evaluable population was used for sensitivity analyses of ORR and included all patients in the all-treated population who had ≥1 adequate post-treatment disease assessment before the start of subsequent anti-cancer therapy.

ORR and 90% two-sided exact CI were calculated for each DLBCL subtype in the all-treated population in part 1 (non-GCB and GCB) and 2 (ABC, GCB, or unclassified). If the lower bound of the CI around the ORR was ≥60% for the ABC DLBCL subtype, then the hypothesis that ORR in the ABC subtype is ≤60% would be rejected at the α = 10% level. DOR was defined as time from first occurrence of CR or PR until first occurrence of recurrent or progressive disease. PFS was defined as time from first study drug administration to disease progression or death. OS was defined as time from first study drug administration until death. Kaplan–Meier methodology was used to estimate DOR distribution for responders and PFS and OS curves, including corresponding quartiles and median.

## Results

### Patients

Overall, 35 patients were enrolled (part 1, 15; part 2, 20). In part 1 (lenalidomide doses: 0 mg, *n* = 3; 15 mg, *n* = 3; 20 mg, *n* = 3; 25 mg, *n* = 6), median age was 58 years (range, 38–89). Median number of prior regimens was three (range, 1–5); 67% of patients were refractory to the last chemotherapy (Table 1); 53% had GCB and 47% had non-GCB DLBCL per IHC. Most patients were categorized as de novo DLBCL (80%); remaining patients were categorized as transformed (13%) or primary mediastinal (7%). The primary reasons for ibrutinib discontinuation included investigator choice (27%) and completed protocol-specified treatment (6 cycles, 27%) (Table 1). Two patients experienced AEs leading to ibrutinib discontinuation. Median time of study was 30.0 months (range, 1.2+ to 34.5). One dose-limiting toxicity (DLT) was reported during the DLT assessment period: 1 grade 5 diffuse alveolar damage at dose level 4. Level 4 was identified as the RP2D (ibrutinib 560 mg, lenalidomide 25 mg, and DA-EPOCH-R).

Twenty-six patients treated at the RP2D were included in the part 2 prespecified analysis of ORR (part 1: 25 mg lenalidomide, *n* = 6; part 2: 25 mg lenalidomide, *n* = 20). Median age was 57.5 years (range, 28–79). Median number of prior regimens was two (range, 1–4); 58% were refractory to the last chemotherapy. When subtype was identified using IHC, 22 patients (85%) had non-GCB, and 3 (12%) had GCB subtype; one patient had no data. One non-GCB patient by IHC had GCB DLBCL by GEP and was then categorized as GCB for further analyses; the other 21 non-GCB patients per IHC were categorized as non-GCB per GEP (14 ABC and 1 unclassified; 6 GEP unavailable) (Table 1). For patients treated at the RP2D, primary reasons for ibrutinib discontinuation included investigator decision (35%) and completion of protocol-specified treatment (6 cycles; 27%) (Table 1). Three patients experienced AEs leading to ibrutinib discontinuation.

All 35 patients (parts 1 and 2) have now withdrawn from the study due to death (50%) and study closure (50%). Median time on study was 19.4 months (range, 0.26+ to 28.22). Eight patients (23%) underwent stem cell transplant following study treatment; this was notable given that the primary study population was transplant-ineligible.

### Safety

**Figure 2.**
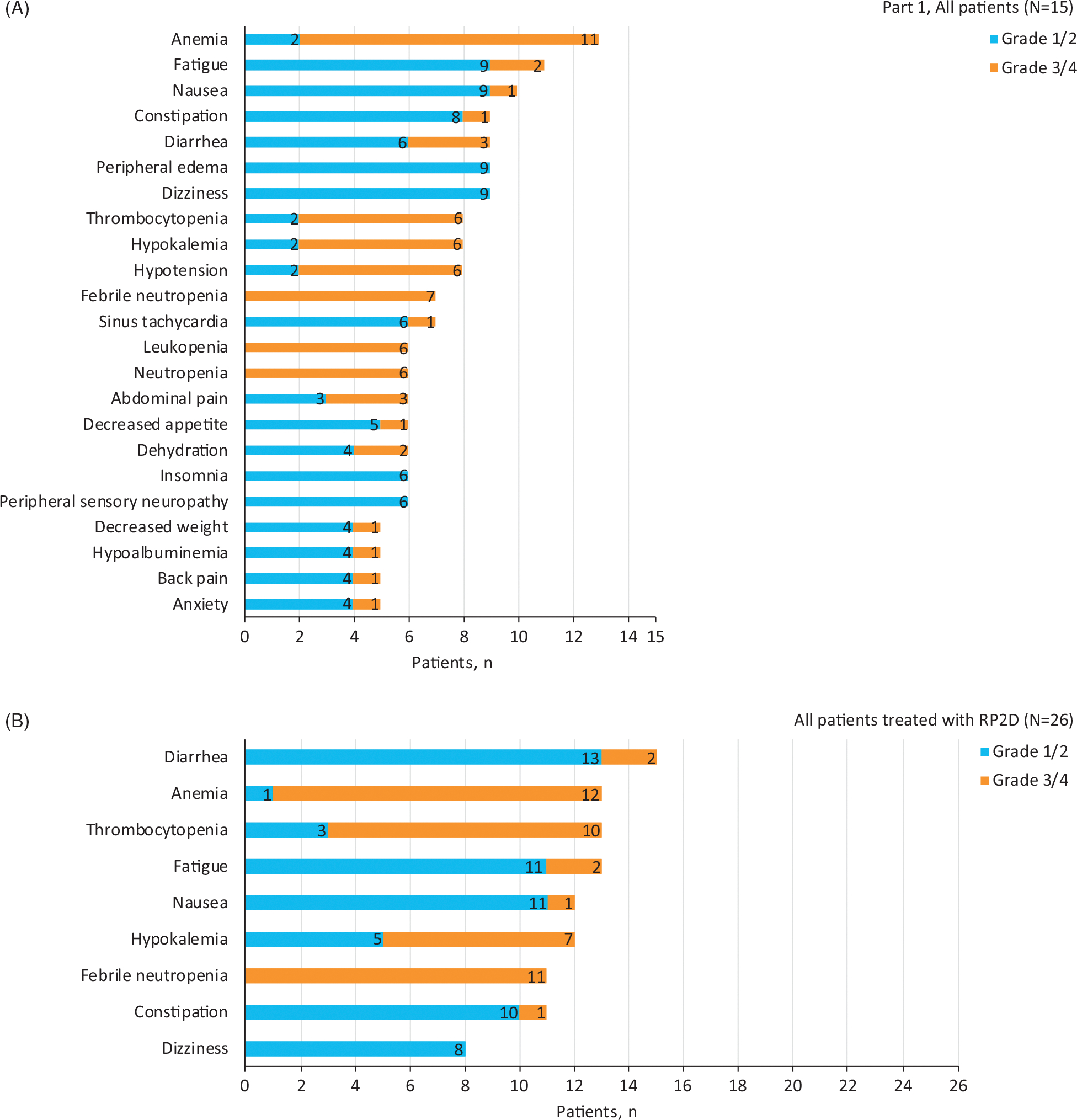
Treatment-emergent adverse events of any grade occurring in ≥30% of the total population in part 1 (A) and in ≥30% of patients treated at the recommended part 2 dose (B). Patients with multiple events for a given preferred term or system organ class are counted once only. Adverse events are sorted by decreasing frequency of preferred term for the total population; the number of patients with each event is shown. RP2D: recommended part 2 dose.

In part 1, all patients (*N* = 15) experienced treatment-emergent AEs (TEAEs), the most common being anemia (87%); fatigue (73%); nausea (67%); constipation, diarrhea, dizziness, and peripheral edema (60% each); and hypokalemia, hypotension, and thrombocytopenia (53% each) (Figure 2(A)). Four patients (27%) experienced atrial fibrillation, and 10 (67%) experienced other cardiac arrhythmias. Most patients (93%) experienced grade ≥3 TEAEs, the most common being anemia (73%); febrile neutropenia (47%); and hypokalemia, leukopenia, neutropenia, and thrombocytopenia (40% each) (Supplementary Table S1). Ten patients (67%) experienced ibrutinib-related TEAEs. Most patients (93%) experienced serious TEAEs, with hypotension (20%), anemia (20%), and febrile neutropenia (20%) being the most common. TEAEs led to ibrutinib discontinuation in 13% of patients and to lenalidomide discontinuation in 7% of patients. There were three fatal TEAEs: gastrointestinal hemorrhage, death, and diffuse alveolar damage (same DLT as described above). Gastrointestinal hemorrhage and death were not related to study drugs; diffuse alveolar damage was considered related to lenalidomide and DA-EPOCH-R.

All patients treated at the RP2D (*n* = 26) experienced TEAEs, the most common being diarrhea (58%), anemia (50%), fatigue (50%), and thrombocytopenia (50%) (Figure 2(B)). Three (12%) patients experienced atrial fibrillation, and eight (31%) experienced other cardiac arrhythmias. No patients experienced cardiomyopathy or cardiac failure. Most common grade ≥3 TEAEs were anemia (46%), febrile neutropenia (42%), thrombocytopenia (38%), and hypokalemia (27%) (Supplementary Table S1). Twenty patients (77%) experienced ibrutinib-related TEAEs. Nineteen patients (73%) experienced serious TEAEs; most common was febrile neutropenia (23%). TEAEs led to ibrutinib and lenalidomide discontinuation in 12% of patients each. Three patients (12%) experienced fatal TEAEs: atrial fibrillation, colitis, and diffuse alveolar damage (same patient from Part 1 treated at RP2D and described previously). Atrial fibrillation was considered related to ibrutinib and occurred in a patient with no previous history of atrial fibrillation; colitis was considered related to DA-EPOCH-R; and diffuse alveolar damage was considered related to lenalidomide and DA-EPOCH-R.

### Response rate and outcome

**Figure 3.**
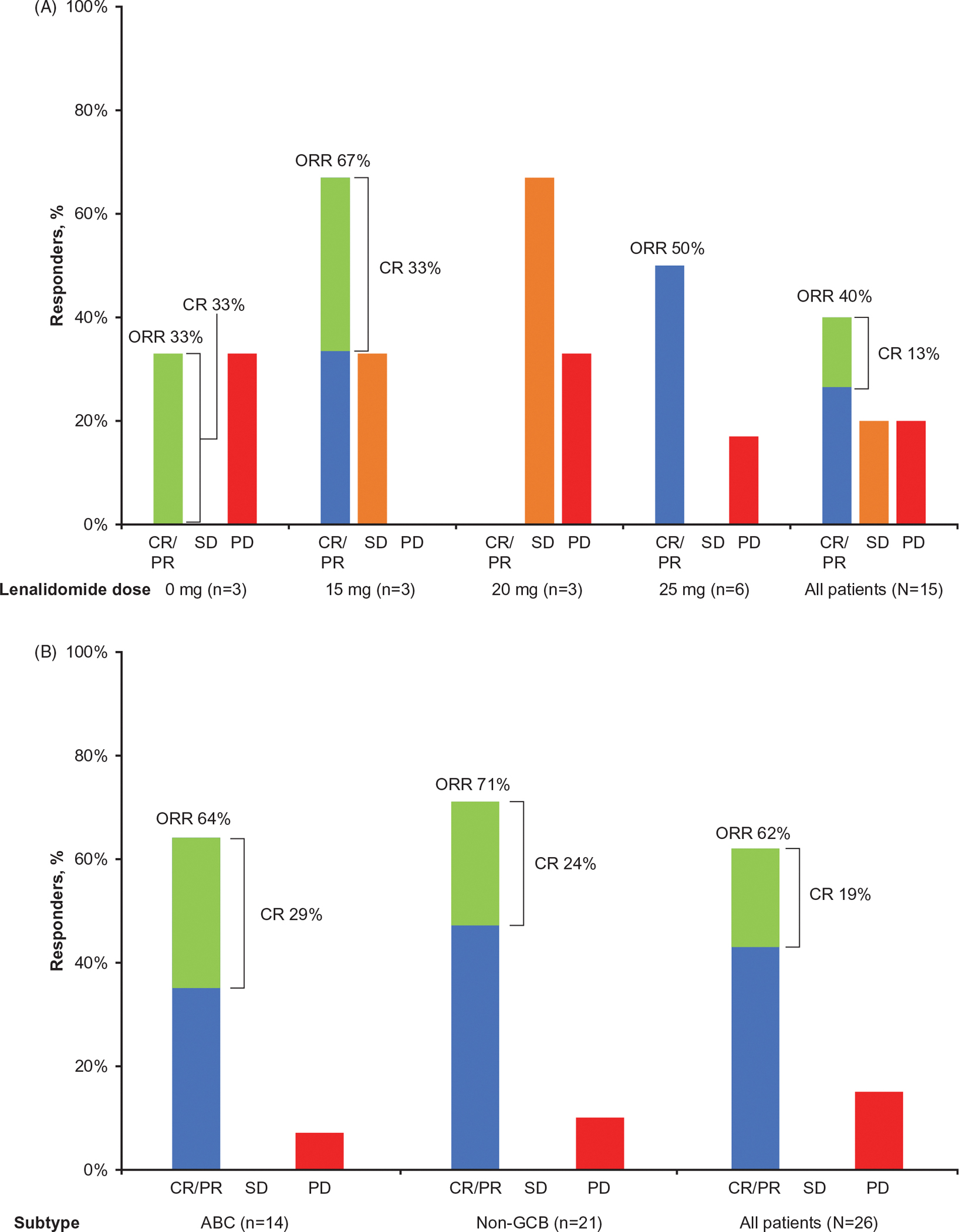
Best overall tumor response in part 1 (A) and all patients treated at recommended part 2 dose (B). ABC: activated B-cell-like per gene expression profiling; CI: confidence interval; CR: complete response; non-GCB: non-germinal center B-cell-like per immunohistochemistry; ORR: overall response rate; PD: progressive disease; PR: partial response; SD: stable disease.

In the all-treated population of part 1 (*N* = 15), ORR was 40% (90% CI, 19.1–64.0; CR, *n* = 2; PR, *n* = 4); three patients (20%) had stable disease (Figure 3(A)). In patients treated at the RP2D (*n* = 26), ORR was 62% (16/26; 90% CI, 43.6–77.4), including 71% (15/21; 90% CI, 51.3–86.8) in non-GCB DLBCL and 64% (*n* = 9/14; 90% CI, 39.0–84.7) in ABC DLBCL (Figure 3(B)). Among patients with ABC DLBCL, 29% (4/14) achieved CR and 36% (5/14) achieved PR. In the response-evaluable population treated at the RP2D (*n* = 20), ORR was 88% (15/17; 90% CI, 67.4–97.9) in non-GCB DLBCL and 90% (9/10; 90% CI, 60.6–99.5) in ABC DLBCL. In responders treated at RP2D (*n* = 16), median DOR was 3.9 months (range, 0.03+ to 10.48) in all patients, 4.3 months (range, 0.03+ to 10.48) in non-GCB DLBCL (*n* = 15), and 4.1 months (range, 0.03+ to 7.69+) in ABC DLBCL (*n* = 9).

**Figure 4.**
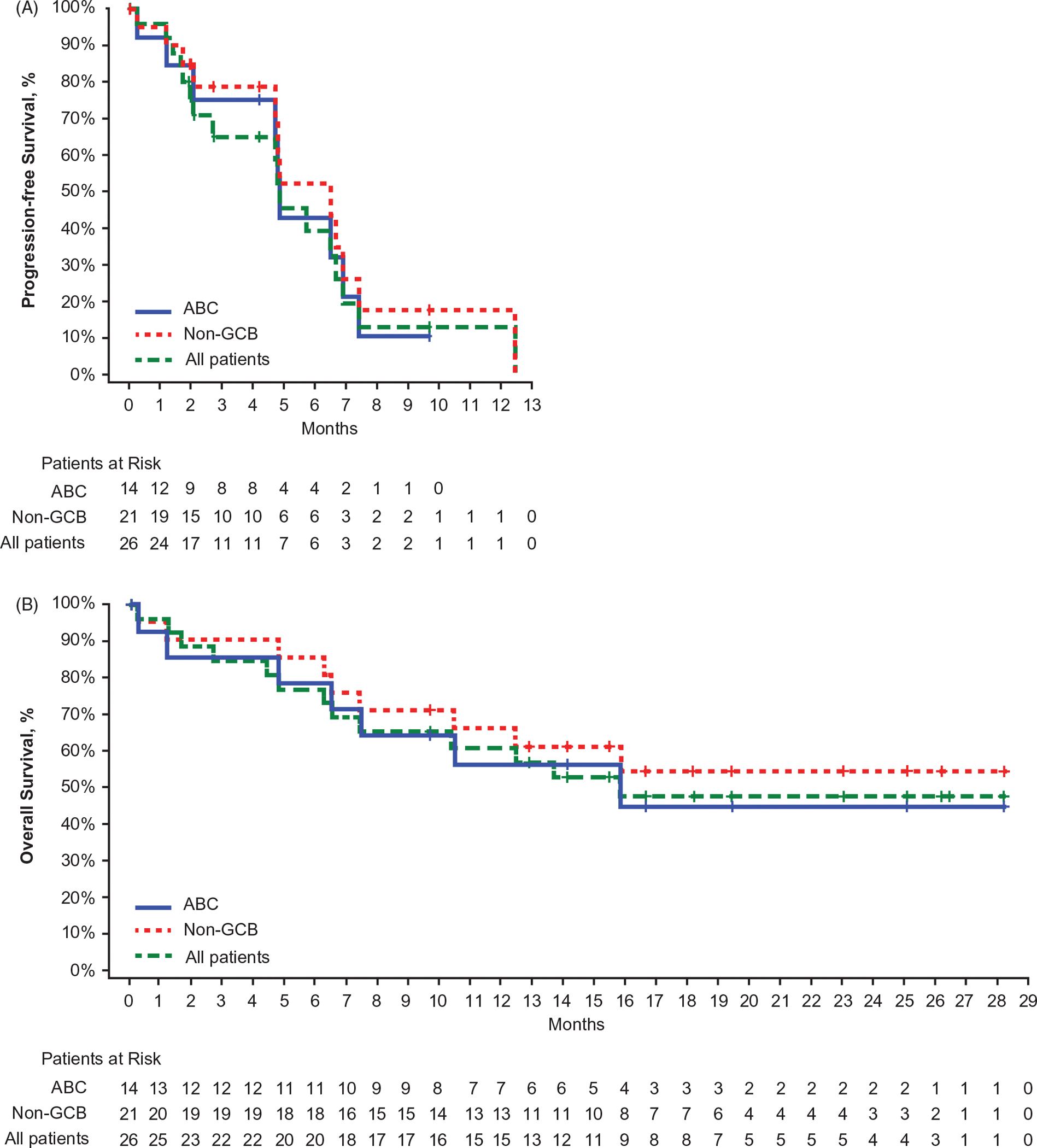
Progression-free survival (A) and overall survival (B) in patients treated at recommended part 2 dose. ABC: activated B-cell-like per gene expression profiling; non-GCB: non-germinal center B-cell-like per immunohistochemistry. Tick marks represent censored patients.

In patients treated at the RP2D (*n* = 26), median PFS was 4.86 months (range, 0.03+ to 12.45) in all patients, 6.51 months (range, 0.03+ to 12.45) in non-GCB DLBCL, and 4.86 months (range, 0.03+ to 9.69+) in ABC DLBCL (Figure 4(A)). Median OS was 15.84 months (range, 0.26 to 28.22+) in all patients, not reached (range, 0.26 to 28.22+) in non-GCB DLBCL, and 15.84 months (range, 0.26 to 28.22+) in ABC DLBCL (Figure 4(B)).

### Pharmacokinetic analyses

Oral ibrutinib at 560 mg/day was rapidly absorbed (median time to maximum concentration [*T*_max_], 2.05 h; mean apparent terminal half-life [*t*_1/2_], 6.98 h). Steady-state levels were achieved on Day 5 of Cycle 1. Mean maximum concentration (*C*_max_; % coefficient of variation) of ibrutinib at steady state was 120 ng/mL (84%); mean steady-state area-under-the-plasma concentration–time curve during the dosing interval (AUC_0–24h_) was 717 ng·h/mL (90%). PCI-45227, an active metabolite of ibrutinib, was detectable at steady-state; mean *t*_1/2_ was 6.74 h. Mean steady-state PCI-45227 *C*_max_ (% coefficient of variation) was 132 ng/mL (44%); AUC_0–24h_ was 1270 ng·h/mL (49%). Mean metabolite-to-parent ratio at steady state was 1.49 for *C*_max_ and 2.26 for AUC_0–24h_.

### Biomarker analyses

Thirty-one of 33 tissue samples were available for DLBCL subtyping; two had inadequate tumor content (Supplementary Table S2). Macrodissection for tumor content enrichment was performed for six samples with <50% tumor tissue. Of the 31 tissue samples (6 macrodissected and 25 total tissue samples), DLBCL subtypes were available for 23 patients: ABC, *n* = 16; GCB, *n* = 6; and unclassified, *n* = 1. The remaining eight tumor tissue samples failed to meet the minimum quality or RNA quantity.

Among the 27 patients treated with ibrutinib and lenalidomide in combination with DA-EPOCH-R who had available molecular data, 25 had subtyping results available. Of the 25 patients with available subtypes, all five CRs were ABC subtype (100%; 5/5); one patient without subtyping available also had a CR. Of the 10 patients with PR, 50% (5/10) had ABC subtype; of the 10 patients who did not achieve an objective response, 60% (6/10) had ABC subtype. Table 2 shows mutations in genes known to discriminate between DLBCL genetic subtypes or to be recurrently mutated in DLBCL. There was a skewing of MYD88 L265P mutations, with a prevalence of 50% (3/6) among patients with a CR versus 19% (4/21) among other patients.

## Discussion

Current treatment options infrequently achieve durable remissions in R/R DLBCL [9,10]. DA-EPOCH-R is a recognized treatment option and a suggested second- or later-line therapy for patients with R/R DLBCL ineligible for transplant [13,14]. In this study, the hypothesis that the addition of ibrutinib and lenalidomide would inhibit BCR signaling and NF-κB in ABC DLBCL and thereby enhance sensitivity to DA-EPOCH-R was assessed. The safety and tolerability of ibrutinib and lenalidomide in combination with DA-EPOCH-R in patients with R/R DLBCL was evaluated. An ibrutinib dose of 560 mg was selected based on a phase 1b study (*n* = 33), in which this dose combined with R-CHOP was deemed safe [29]. In part 1, patients received DA-EPOCH-R and ibrutinib 560 mg with escalating doses of lenalidomide. As the MTD was not reached in part 1, patients received standard doses of DA-EPOCH-R, ibrutinib 560 mg, and lenalidomide 25 mg as the RP2D. Ibrutinib-related TEAEs occurred in approximately three-fourths of patients, the most common being consistent with known ibrutinib AEs, specifically diarrhea (35%) and fatigue (27%) [30,31]. Consistent with known ibrutinib and lenalidomide AEs, 31% (*n* = 8) of patients receiving the RP2D experienced cardiac arrhythmias, and 12% (*n* = 3) experienced atrial fibrillation. The rate of grade ≥3 febrile neutropenia (42%) was higher than rates reported in trials with newer therapies for DLBCL (6–10%) [32–34]. In patients treated at the RP2D, TEAEs led to ibrutinib and lenalidomide discontinuation in 12% (3/26) of patients each. These results are somewhat expected, given the heavily pretreated patient population and the intensity of the multidrug combination being investigated [35,36]. The large (*N* = 838) PHOENIX phase 3 study showed that ibrutinib 560 mg plus R-CHOP for 6–8 cycles led to more serious AEs and R-CHOP discontinuation in patients ≥60 years of age, suggesting that ibrutinib combined with R-CHOP is not tolerable at this dose and in this particular setting [23] and demonstrating the difficulties of combining chemotherapy regimens with targeted agents.

Previous reports have shown that ABC tumor cells, unlike GCB, are dependent on activation of the NF-κB pathway for survival [37]. The addition of ibrutinib and lenalidomide to DA-EPOCH-R was hypothesized to inhibit BCR signaling and NF-κB activation, thus improving the outcomes of patients with R/R ABC DLBCL. In part 2, enrollment was limited to patients with non-GCB DLBCL; these patients were later classified as ABC, GCB, and unclassified subtypes by GEP. The success criterion for this study was based on historical data and set at an ORR of 60% [13,38]. The ORR was 71% in all patients with non-GCB DLBCL at the RP2D and 64% in the subset with ABC subtype. In the response-evaluable population, ORR was 90% in the ABC subset and 88% in the non-GCB subset. However, only 14/26 patients who received the RP2D had ABC DLBCL, and the study was not powered to test the hypothesis per the original sample size. Because of low enrollment, the study was terminated early. Notably, 8/35 (23%) patients underwent stem cell transplant following study treatment, despite being transplant-ineligible at enrollment.

The pharmacokinetics of ibrutinib in this combination regimen were similar to those reported with single-agent ibrutinib. In line with observations in patients with marginal zone lymphoma or mantle cell lymphoma receiving single-agent ibrutinib 560 mg/day, ibrutinib was detected in plasma at steady state (Cycle 1, Day 5) [30,31]. Absorption of ibrutinib was rapid (*T*_max,_ 2.05 h) and similar to that reported previously with single-agent ibrutinib (*T*_max_, 1–2 h) [30,31]. Mean steady-state AUC_0–24h_ (% coefficient of variation; 717 ng·h/mL [90%]) was also comparable to that observed at the ibrutinib 560 mg dose in patients with mantle cell (865 ng·h/mL [69%]) and marginal zone lymphoma (978 ng·h/mL [82%]) [30,31].

Molecular analysis of tumors was consistent with the expected synergy of ibrutinib and lenalidomide in molecular subtypes of DLBCL. There were 6 CRs in patients treated with ibrutinib and lenalidomide plus DA-EPOCH-R: 1 without subtyping results and five in the ABC subtype, consistent with previous work demonstrating that ibrutinib and lenalidomide synergize in killing cell lines in models of ABC but not GCB DLBCL [39]. Importantly, these results conform with the pre-study hypothesis that response to regimens containing ibrutinib and lenalidomide would be more favorable in patients with ABC versus non-ABC DLBCL. Recent genetic profiling of DLBCL tumors subdivided ABC DLBCL into four subtypes: MCD, BN2, A53, and N1 [28]. The synergy of ibrutinib and lenalidomide was specifically observed in models of MCD DLBCL, which is characterized by the MYD88 L265P mutation and/or a CD79B ITAM motif mutation in 84% of cases [28]. Three of six CRs had the MYD88 L265P mutation; one belonged to the MCD subtype due to a CD79B mutation and several other mutations typical of MCD (*PIM1*, *HLA-B*, and *BTG1*). Several tumors with the MYD88 L265P mutation did not respond to ibrutinib, lenalidomide, and DA-EPOCH-R, suggesting that additional genetic and/or epigenetic attributes of DLBCL tumors may influence response to this regimen.

To assess whether the combination of ibrutinib and lenalidomide with DA-EPOCH-R has a benefit over DA-EPOCH-R alone, a randomized study with large patient numbers would be needed. In this study, too few patients were enrolled to assess whether the addition of ibrutinib and lenalidomide to DA-EPOCH-R had a significant effect on outcomes. While the ORR was higher than previously reported with DA-EPOCH-R alone (68%), the combination of ibrutinib, lenalidomide, and DA-EPOCH-R was less tolerable [13,14]. Despite the more recent successes of CAR T-cell therapy, a paucity of published trials with positive outcomes and the lack of standard of care in transplant-ineligible R/R DLBCL highlights the need for more effective, less toxic therapies. Investigational regimens have shown promise in transplant-ineligible patients with DLBCL in early phase trials: the combination of polatuzumab vedotin, bendamustine, and rituximab achieved a CR rate of 40%, and tafasitamab plus lenalidomide achieved a CR rate of 43% [32,34].

In conclusion, despite a high number of individual drug components in this regimen, the combination of ibrutinib, lenalidomide, and DA-EPOCH-R had a manageable safety profile in this patient population. In addition, ibrutinib 560 mg and lenalidomide 25 mg on Days 1–7 in combination with standard doses of DA-EPOCH-R on Days 1–5 of a 21-day cycle demonstrated evidence of antitumor activity in patients with R/R DLBCL.

## Supplementary Material

33856277_WyndhamWilson_SuppleMaterial

## Figures and Tables

**Table 1.** Baseline characteristics and patient disposition in study part 1 and in patients treated at the recommended part 2 dose.

	Part 1 All patients *N* = 15	All patients treated at RP2D		
		ABC *n* = 14	Non-GCB *n* = 21	Total *n* = 26

Median age (range), years	58 (38–89)	65 (28–79)	60 (28–79)	57.5 (28–79)
Sex, *n* (%)				
Male	13 (87)	9 (64)	14 (67)	18 (69)
Female	2 (13)	5 (36)	7 (33)	8 (31)
Race, *n* (%)				
Asian	1 (7)	1 (7)	1 (5)	1 (4)
Black or African American	0	2 (14)	2 (10)	2 (8)
White	14 (93)	10 (71)	17 (81)	21 (81)
Unknown	0	1 (7)	1 (5)	2 (8)
DLBCL category, *n* (%)				
*De novo*	12 (80)	14 (100)	21 (100)	24 (92)
Transformed	2 (13)	0	0	1 (4)
Primary mediastinal	1 (7)	0	0	1 (4)
DLBCL subtype per IHC by local review, *n* (%)				
GCB	8 (53)	0	0	3 (12)
Non-GCB	7 (47)	14 (100)	21 (100)	22 (85)
Not reported/missing	0	0	0	1 (4)
Number of prior systemic therapies for DLBCL				
Median (range)	3 (1 –5)	2.5 (1 –4)	2 (1 –4)	2 (1 –4)
Disease status after last treatment before study				
Relapsed	5 (33)	6 (43)	9 (43)	11 (42)
Refractory	10 (67)	8 (57)	12 (57)	15 (58)
Ann Arbor staging, *n* (%)				
I	0	0	1 (5)	1 (4)
IE	2 (13)	0	0	0
II	0	3 (21)	5 (24)	5 (19)
IIE	0	0	1 (5)	1 (4)
III	4 (27)	3 (21)	4 (19)	8 (31)
IIIE	0	0	0	0
IIIE, S	0	1 (7)	1 (5)	1 (4)
IV	9 (60)	5 (36)	7 (33)	8 (31)
Not reported	0	2 (14)	2 (10)	2 (8)
Bulky disease, *n* (%)				
Present	11 (73)	9 (64)	12 (57)	14 (54)
5–10 cm	8 (53)	6 (43)	9 (43)	11 (42)
>10 cm	3 (20)	3 (21)	3 (14)	3 (12)
Not present	3 (20)	5 (36)	9 (43)	11 (42)
Not reported	1 (7)	0	0	1 (4)
Discontinued ibrutinib, *n* (%)	15 (100)	14 (100)	21 (100)	26 (100)
Confirmed disease progression	3 (20)	1 (7)	2 (10)	4 (15)
Death	2 (13)	0	0	1 (4)
Intercurrent illness or AE	2 (13)	3 (21)	3 (14)	3 (12)
Patient withdrew from study	0	1 (7)	2 (10)	2 (8)
Investigator decision^a^	4 (27)	4 (29)	8 (38)	9 (35)
Completion of treatment (6 cycles)	4 (27)	5 (36)	6 (29)	7 (27)
Discontinued lenalidomide, *n* (%)	12 (80)	14 (100)	21 (100)	26 (100)
Confirmed disease progression	2 (13)	1 (7)	2 (10)	4 (15)
Death	1 (7)	0	0	1 (4)
Intercurrent illness or AE	1 (7)	3 (21)	3 (14)	3 (12)
Patient withdrew from study	0	1 (7)	2 (10)	2 (8)
Investigator decision	4 (27)	4 (29)	8 (38)	9 (35)
Completion of treatment (six cycles)	4 (27)	5 (36)	6 (29)	7 (27)
Received DA-EPOCH-R, exited study, *n* (%)	15 (100)	14 (100)	21 (100)	26 (100)
Study terminated by sponsor	6 (40)	7 (50)	12 (57)	13 (50)
Death	9 (60)	7 (50)	9 (43)	13 (50)

ABC: activated B-cell-like; AE: adverse event; DA-EPOCH-R: dose-adjusted ibrutinib, lenalidomide, and dose-adjusted cyclophosphamide, doxorubicin, etoposide, vincristine, and prednisone, with or without rituximab; DLBCL: diffuse large B-cell lymphoma; GCB: germinal center B-cell-like; non-GCB: non-germinal center B-cell-like; RP2D: recommended part 2 dose.

a Reasons for the investigator decision to withdraw patients were patient-pursued transplant (*n* = 5), best interest of patient (*n* = 2), toxicity concerns despite achievement of CR (*n* = 1), failure to achieve CR after three cycles (*n* = 1), lesions remained metabolically stable after three cycles (*n* = 1), and unconfirmed PD (*n* = 1).

**Table 2.** Hallmark genetic mutations by DLBCL subtype and response.

Patient	Lenalidomide dose with DA-EPOCH-R + ibrutinib^a^	Response	Cell-of-origin assignment per IHC	Gene expression subgroup	Hallmark genetic mutations^b^

1	25	CR	Non-GCB	ABC	ARID1A_Q2176fs; MYD88_L265P; TBL1XR1_S447R; TP53_F134C
2	25	CR	Non-GCB	ABC	KMT2D_L561X
3	25	CR	Non-GCB	ND	TET2_E1106fs
4	25	CR	Non-GCB	ABC	ARID1A_R1528X; CD58_K57fs; KMT2D_Q3499X; CREBBP_Y1503N
5	25	CR	Non-GCB	ABC	CD79B_Y196H; MYD88_L265P; PIM1_G139D; HLA-B_G50D; BTG1_L37L
6	15	CR	Non-GCB	ABC	MYD88_L265P; BTG2_A45T; GRHPR_G48S; OSBPL12_S16T; PIM1_G190D;
7	15	PR	Non-GCB	GCB	KMT2D_Y2199fs; MYD88_L265P; NFKBIA_Q44X; NOTCH2_R2400X; TP53_G245D
8	25	PR	Non-GCB	ABC	BCL6_G559R; CDKN2A_R80X; IRF8_N87Y; PIM1_L93F
9	25	PR	Non-GCB	Unclassified	CREBBP_I1084fs; KMT2D_R5048H
10	25	PR	Non-GCB	ABC	CD79B_Splice; HLA-A_Q139X; SPEN_S1103X; BTG2_S31N; GRHPR_G48S; HLA-C_G144D
11	25	PR	Non-GCB	ABC	UBE2A_Y82D
12	25	PR	Non-GCB	ND	B2M_M1?; EZH2_Y641H
13	25	PR	Non-GCB	ABC	KMT2D_W1591X; NFKBIA_Q44X
14	25	PR	Non-GCB	Unclassified	SPEN_Q1250X; CD70_R100C; TMEM30A_S280fs; PRKCB_A94V;TET2_Q321X
15	25	PR	Non-GCB	GCB	NOTCH1_Q2501X; SPEN_R1265X
16	25	PR	Non-GCB	GCB	BCL2_L119L; IRF8_T80A; TP53_C176W; FOXC1_Q2X
17	25	PR	Non-GCB	ABC	PRDM1_Q225X; TAP1_Splice; TP53_G245S; KMT2D_Q3599X
18	20	SD	GCB	GCB	BCL10_L209X; EZH2_Y641F; MEF2B_D83V
19	20	SD	GCB	GCB	ARID1A_Q405X; BCL2_L23L; MEF2B_Y69H
20	25	PD	Non-GCB	ABC	BCL10_K146fs; CD58_K184fs; CD79B_Y196H; KMT2D_R4484X; TET2_Q1903X; IRF8_Q371X
21	25	PD	GCB	GCB	KMT2D_E1254X; TET2_V1371fs; TP53_Y234C
22	25	PD	Non-GCB	ABC	ARID1A_K1815fs; CREBBP_Q249X; KMT2D_R4484X; MYD88_L265P; NOTCH2_Q2140X; SETD1B_Q1045X; TNFAIP3_T118fs; TET2_Q939X
23	25	non-CR/PR	Non-GCB	ABC	CREBBP_L431P; MYD88_S243N; TP53_S215G
24	25	non-CR/PR	Non-GCB	ABC	HLA-B_C227Y; MYD88_L265P; HLA-B_C227Y; PIM1_S77N
25	25	non-CR/PR	Non-GCB	ABC	MYD88_L265P
26	25	non-CR/PR	Non-GCB	ABC	CARD11_F115I; CD58_R152X; KMT2D_G2794fs; NOTCH1_P2514fs
27	25	non-CR/PR	Non-GCB	GCB	None detected

CR: complete response; DA-EPOCH-R: dose-adjusted etoposide, prednisone, vincristine, cyclophosphamide, doxorubicin, and rituximab; DLBCL: diffuse large B-cell lymphoma; ND: not determined; non-CR/PR: no response clinically, but without radiologic scan evidence; PD: progressive disease; PR: partial response; SD: stable disease; WES: whole exome sequencing.

a Does not include three patients treated with DA-EPOCH-R plus ibrutinib (lenalidomide 0 mg); of these three patients, one had a CR, one had PD, and one had no response assessment (PFS, 1.6 months).

b Mutation calls were more frequent from RNA-seq data than from WES data, consistent with a higher percentage of false-positive calls using RNA-seq data. Therefore, as a quality control measure, only mutations that were confirmed on both RNA-seq and WES or that have been recurrently identified in DLBCL tumors are displayed.

## Data Availability

Requests for access to individual participant data from clinical studies conducted by Pharmacyclics LLC, an AbbVie Company, can be submitted through Yale Open Data Access (YODA) Project site at http://yoda.yale.edu.

## References

[1] Howlader N, Noone AM, Krapcho M, SEER Cancer Statistics Review 1975–2014. Bethesda (MD): National Cancer Institute.

[2] Lenz G, Staudt LM. Aggressive lymphomas. N Engl J Med. 2010;362(15):1417–1429. 20393178 10.1056/NEJMra0807082 PMC7316377

[3] Coiffier B, Thieblemont C, Van Den Neste E, Long-term outcome of patients in the LNH-98.5 trial, the first randomized study comparing rituximab-CHOP to standard CHOP chemotherapy in DLBCL patients: a study by the Groupe d’Etudes des Lymphomes de l’Adulte. Blood. 2010;116(12):2040–2045. 20548096 10.1182/blood-2010-03-276246 PMC2951853

[4] Pfreundschuh M, Trumper L, Osterborg A, CHOP-like chemotherapy plus rituximab versus CHOP-like chemotherapy alone in young patients with good-prognosis diffuse large-B-cell lymphoma: a randomised controlled trial by the MabThera International Trial (MInT) Group. Lancet Oncol. 2006;7(5):379–391. 16648042 10.1016/S1470-2045(06)70664-7

[5] Wilson WH, Dunleavy K, Pittaluga S, Phase II study of dose-adjusted EPOCH and rituximab in untreated diffuse large B-cell lymphoma with analysis of germinal center and post-germinal center biomarkers. J Clin Oncol. 2008;26(16):2717–2724. 18378569 10.1200/JCO.2007.13.1391 PMC2409217

[6] Purroy N, Bergua J, Gallur L, Long-term follow-up of dose-adjusted EPOCH plus rituximab (DA-EPOCH-R) in untreated patients with poor prognosis large B-cell lymphoma. A phase II study conducted by the Spanish PETHEMA Group. Br J Haematol. 2015;169(2):188–198. 25521006 10.1111/bjh.13273

[7] Wilson WH, Jung SH, Porcu P, A Cancer and Leukemia Group B multi-center study of DA-EPOCH-rituximab in untreated diffuse large B-cell lymphoma with analysis of outcome by molecular subtype. Haematologica. 2012;97(5):758–765. 22133772 10.3324/haematol.2011.056531 PMC3342980

[8] Sehn LH, Donaldson J, Chhanabhai M, Introduction of combined CHOP plus rituximab therapy dramatically improved outcome of diffuse large B-cell lymphoma in British Columbia. J Clin Oncol. 2005;23(22):5027–5033. 15955905 10.1200/JCO.2005.09.137

[9] Crump M, Neelapu SS, Farooq U, Outcomes in refractory diffuse large B-cell lymphoma: results from the international SCHOLAR-1 study. Blood. 2017;130(16):1800–1808. 28774879 10.1182/blood-2017-03-769620 PMC5649550

[10] Gisselbrecht C, Glass B, Mounier N, Salvage regimens with autologous transplantation for relapsed large B-cell lymphoma in the rituximab era. J Clin Oncol. 2010;28(27):4184–4190. 20660832 10.1200/JCO.2010.28.1618 PMC3664033

[11] Zelenetz AD, Hamlin P, Kewalramani T, Ifosfamide, carboplatin, etoposide (ICE)-based second-line chemotherapy for the management of relapsed and refractory aggressive non-Hodgkin’s lymphoma. Ann Oncol. 2003;14 (1):i5–i10. 12736224 10.1093/annonc/mdg702

[12] Mey UJ, Orlopp KS, Flieger D, Dexamethasone, high-dose cytarabine, and cisplatin in combination with rituximab as salvage treatment for patients with relapsed or refractory aggressive non-Hodgkin’s lymphoma. Cancer Invest. 2006;24(6):593–600. 16982464 10.1080/07357900600814490

[13] Jermann M, Jost LM, Taverna C, Rituximab-EPOCH, an effective salvage therapy for relapsed, refractory or transformed B-cell lymphomas: results of a phase II study. Ann Oncol. 2004;15(3):511–516. 14998858 10.1093/annonc/mdh093

[14] Wilson WH, Gutierrez M, O’Connor P, The role of rituximab and chemotherapy in aggressive B-cell lymphoma: a preliminary report of dose-adjusted EPOCH-R. Semin Oncol. 2002;29(1S2):41–47. 10.1053/sonc.2002.30151 28140091

[15] Bao F, Wan W, He T, Autologous CD19-directed chimeric antigen receptor-T cell is an effective and safe treatment to refractory or relapsed diffuse large B-cell lymphoma. Cancer Gene Ther. 2019;26(7–8):248–255. 30622321 10.1038/s41417-018-0073-7

[16] Neelapu SS, Locke FL, Bartlett NL, Axicabtagene ciloleucel CAR T-cell therapy in refractory large B-cell lymphoma. N Engl J Med. 2017;377(26):2531–2544. 29226797 10.1056/NEJMoa1707447 PMC5882485

[17] Wilson WH, Young RM, Schmitz R, Targeting B cell receptor signaling with ibrutinib in diffuse large B cell lymphoma. Nat Med. 2015;21(8):922–926. 26193343 10.1038/nm.3884 PMC8372245

[18] Hernandez-Ilizaliturri FJ, Deeb G, Zinzani PL, Higher response to lenalidomide in relapsed/refractory diffuse large B-cell lymphoma in nongerminal center B-cell-like than in germinal center B-cell-like phenotype. Cancer. 2011;117(22):5058–5066. 21495023 10.1002/cncr.26135

[19] Witzig TE, Vose JM, Zinzani PL, An international phase II trial of single-agent lenalidomide for relapsed or refractory aggressive B-cell non-Hodgkin’s lymphoma. Ann Oncol. 2011;22(7):1622–1627. 21228334 10.1093/annonc/mdq626

[20] Davis RE, Ngo VN, Lenz G, Chronic active B-cell-receptor signalling in diffuse large B-cell lymphoma. Nature. 2010;463(7277):88–92. 20054396 10.1038/nature08638 PMC2845535

[21] Honigberg LA, Smith AM, Sirisawad M, The Bruton tyrosine kinase inhibitor PCI-32765 blocks B-cell activation and is efficacious in models of auto-immune disease and B-cell malignancy. Proc Natl Acad Sci USA. 2010;107(29):13075–13080. 20615965 10.1073/pnas.1004594107 PMC2919935

[22] Sauter CS, Matasar MJ, Schoder H, A phase 1 study of ibrutinib in combination with R-ICE in patients with relapsed or primary refractory DLBCL. Blood. 2018;131(16):1805–1808. 29386196 10.1182/blood-2017-08-802561 PMC5909762

[23] Younes A, Sehn LH, Johnson P, Randomized phase III trial of ibrutinib and rituximab plus cyclophosphamide, doxorubicin, vincristine, and prednisone in non-germinal center B-cell diffuse large B-cell lymphoma. J Clin Oncol. 2019;37(15):1285–1295. 30901302 10.1200/JCO.18.02403 PMC6553835

[24] Vitolo U, Witzig TE, Gascoyne RD, ROBUST: first report of phase III randomized study of lenalidomide/R-CHOP (R2 -CHOP) vs placebo/R-CHOP in previously untreated ABC-type diffuse large B-cell lymphoma. Hematol Oncol. 2019;37(S2):36–37.

[25] Cheson BD. The International Harmonization Project for response criteria in lymphoma clinical trials. Hematol Oncol Clin North Am. 2007;21(5):841–854. 17908623 10.1016/j.hoc.2007.06.011

[26] Hans CP, Weisenburger DD, Greiner TC, Confirmation of the molecular classification of diffuse large B-cell lymphoma by immunohistochemistry using a tissue microarray. Blood. 2004;103(1):275–282. 14504078 10.1182/blood-2003-05-1545

[27] Scott DW, Wright GW, Williams PM, Determining cell-of-origin subtypes of diffuse large B-cell lymphoma using gene expression in formalin-fixed paraffin-embedded tissue. Blood. 2014;123(8):1214–1217. 24398326 10.1182/blood-2013-11-536433 PMC3931191

[28] Schmitz R, Wright GW, Huang DW, Genetics and pathogenesis of diffuse large B-cell lymphoma. N Engl J Med. 2018;378(15):1396–1407. 29641966 10.1056/NEJMoa1801445 PMC6010183

[29] Younes A, Thieblemont C, Morschhauser F, Combination of ibrutinib with rituximab, cyclophosphamide, doxorubicin, vincristine, and prednisone (R-CHOP) for treatment-naive patients with CD20-positive B-cell non-Hodgkin lymphoma: a non-randomised, phase 1b study. Lancet Oncol. 2014;15(9):1019–1026. 25042202 10.1016/S1470-2045(14)70311-0

[30] Imbruvica (ibrutinib) [package insert]. Sunnyvale (CA): Pharmacyclics LLC; 2020.

[31] Imbruvica 140 mg hard capsules [summary of product characteristics]. London (UK): European Medicines Agency; 2014.

[32] Sehn LH, Herrera AF, Flowers CR, Polatuzumab vedotin in relapsed or refractory diffuse large B-cell lymphoma. J Clin Oncol. 2020;38(2):155–165. 31693429 10.1200/JCO.19.00172 PMC7032881

[33] Hamadani M, Radford J, Carlo-Stella C, Final results of a phase 1 study of loncastuximab tesirine in relapsed/refractory B-cell non-Hodgkin lymphoma. Blood. 2020;blood.2020007512. 10.1182/blood.2020007512 PMC8138546 33211842

[34] Salles G, Duell J, González Barca E, Tafasitamab plus lenalidomide in relapsed or refractory diffuse large B-cell lymphoma (L-MIND): a multicentre, prospective, single-arm, phase 2 study. Lancet Oncol. 2020;21(7):978–988. 32511983 10.1016/S1470-2045(20)30225-4

[35] Rule S, Dreyling M, Goy A, Ibrutinib for the treatment of relapsed/refractory mantle cell lymphoma: extended 3.5-year follow up from a pooled analysis. Haematologica. 2019;104(5):e211–e214. 30442728 10.3324/haematol.2018.205229 PMC6518912

[36] Byrd JC, Furman RR, Coutre S, Ibrutinib treatment for first-line and relapsed/refractory chronic lymphocytic leukemia: final analysis of the pivotal phase Ib/II PCYC-1102 study. Clin Cancer Res. 2020;26(15):3918–3927. 32209572 10.1158/1078-0432.CCR-19-2856 PMC8175012

[37] Davis RE, Brown KD, Siebenlist U, Constitutive nuclear factor kappaB activity is required for survival of activated B cell-like diffuse large B cell lymphoma cells. J Exp Med. 2001;194(12):1861–1874. 11748286 10.1084/jem.194.12.1861 PMC2193582

[38] Gutierrez M, Chabner BA, Pearson D, Role of a doxorubicin-containing regimen in relapsed and resistant lymphomas: an 8-year follow-up study of EPOCH. J Clin Oncol. 2000;18(21):3633–3642. 11054436 10.1200/JCO.2000.18.21.3633

[39] Yang Y, Shaffer AL III, Emre NC, Exploiting synthetic lethality for the therapy of ABC diffuse large B cell lymphoma. Cancer Cell. 2012;21(6):723–737. 22698399 10.1016/j.ccr.2012.05.024 PMC4059833

