# Supplementary material for: Phase 1b/2 study of ibrutinib and lenalidomide with dose-adjusted EPOCH-R in patients with relapsed/refractory diffuse large B-cell lymphoma
Source: Leuk Lymphoma. Author manuscript; Available in PMC 2023 Feb 8. (PMC9907362; doi:10.1080/10428194.2021.1907371)
Supplement: 33856277_WyndhamWilson_SuppleMaterial [file NIHMS1864024-supplement-33856277_WyndhamWilson_SuppleMaterial.pdf]

Supplementary Material

Supplementary Text S1: Dose adjustment

For hematologic toxicities of thrombocytopenia and neutropenia, the lenalidomide dose was held for the remainder of the cycle and then reduced by 1 dose level (−5 mg) at day 1 of the next cycle if the patient met criteria to initiate that cycle; 5 mg was the minimum dose, after which treatment would be discontinued.

Supplementary Text S2: Endpoints and assessments

Lesions not well visualized by computed tomography were measured at baseline and until disease progression by magnetic resonance imaging. Patients were restaged after cycles 3 and 6 of treatment, then every 3, 4, and 6 months during posttreatment in years 1, 2, and 3, respectively. After disease progression, survival status was assessed approximately every 12 weeks until death, study withdrawal, loss to follow-up, or sponsor-termination of the study, whichever came first.

Supplementary Text S3: DLBCL subtyping

Personalis Laboratories (Menlo Park, CA) extracted RNA from all samples using the AllPrep DNA/RNA FFPE kit (Qiagen, Hilden, Germany). RNA was then analyzed using the Lymph2Cx subtyping assay on the NanoString platform (Seattle, WA). In the Lymph2Cx assay, 15 test genes and five housekeeping genes (for normalization), selected based on their ability to accurately replicate the cell-of-origin assignment model of Lenz et al, differentiated GCB and

ABC subtypes.[1] DLBCL subtype was derived from a linear predictor score based on the relative expression of test genes.[2]

Supplementary Table S1. Treatment-emergent adverse events of grade ≥3 occurring in ≥10% of patients treated at the recommended part 2 dose

	Part 1					All patients treated at RP2D N=26
	Enrolled at Lenalidomide Dose				All patients, N=15	
	0 mg n=3	15 mg n=3	20 mg n=3	25 mg n=6		
Anemia	2 (67)	2 (67)	2 (67)	5 (83)	11 (73)	12 (46)
Febrile neutropenia	1 (33)	1 (33)	1 (33)	4 (67)	7 (47)	11 (42)
Thrombocytopenia	2 (67)	1 (33)	1 (33)	2 (33)	6 (40)	10 (39)
Hypokalemia	0	2 (67)	1 (33)	3 (50)	6 (40)	7 (27)
Neutropenia	2 (67)	1 (33)	2 (67)	1 (17)	6 (40)	5 (19)
Leukopenia	1 (33)	2 (67)	1 (33)	2 (33)	6 (40)	4 (15)
Decreased platelet count	0	1 (33)	1 (33)	1 (17)	3 (20)	4 (15)

RP2D: recommended part 2 dose; TEAE: treatment-emergent adverse event.

Patients with multiple events for a given preferred term or system organ class are counted once.

Adverse events are sorted by decreasing frequency of preferred term for all patients treated at RP2D.

Supplementary Table S2. Diffuse large B-cell lymphoma subtype analysis by gene expression profiling

**Local IHC**	**Macrodissection**	**Lymph2Cx Prediction**
Non-GCB (n=26)	Yes (n=5)	ABC (n=2)
		GCB (n=2)
		N/A (n=1)
	No (n=21)	ABC (n=14)
		N/A (n=6)
		Unclassified (n=1)
GCB (n=7)[a]	Yes (n=1)	N/A (n=1)
	No (n=4)	GCB (n=4)

[a]Includes 2 patients with GCB subtype who did not have tumor tissue available for further analysis.

ABC: activated B-cell–like; IHC: immunohistochemistry; GCB: germinal center B-cell–like; non-GCB: non-germinal center B-cell–like; N/A: data not available due to inadequate tumor content or test sample failing quantity and/or quality of assay requirement.

Local IHC: Local pathology laboratories performed the IHC assay to determine DLBCL subtypes and the result was submitted to the study.

Macrodissection: some samples were macrodissected to enrich for tumor content.

Lymph 2Cx prediction: A linear prediction score generated by the Lymph 2Cx algorithm was used to predict the likelihood of an ABC subtype.

References

1. Lenz G, Wright G, Dave SS, et al. Stromal gene signatures in large-B-cell lymphomas. *N Engl J Med*. 2008;359(22):2313–2323.
2. Scott DW, Wright GW, Williams PM, et al. Determining cell-of-origin subtypes of diffuse large B-cell lymphoma using gene expression in formalin-fixed paraffin-embedded tissue. *Blood*. 2014;123(8):1214–1217.
